# Uveitis Is a Risk Factor for Juvenile Idiopathic Arthritis' Significant Flare in Patients Treated With Biologics

**DOI:** 10.3389/fped.2022.849940

**Published:** 2022-06-15

**Authors:** Mikhail M. Kostik, Ekaterina V. Gaidar, Lubov S. Sorokina, Ilya S. Avrusin, Tatiana N. Nikitina, Eugenia A. Isupova, Irina A. Chikova, Yuri Yu. Korin, Elizaveta D. Orlova, Ludmila S. Snegireva, Vera V. Masalova, Margarita F. Dubko, Olga V. Kalashnikova, Vyacheslav G. Chasnyk

**Affiliations:** ^1^Saint-Petersburg State Pediatric Medical University, Saint Petersburg, Russia; ^2^Pediatric Research and Clinical Center for Infection Diseases, Saint Petersburg, Russia

**Keywords:** juvenile idiopathic arthritis, uveitis, flare, adalimumab, methotrexate

## Abstract

**Objectives:**

Uveitis is the most frequent extra-articular manifestation of juvenile idiopathic arthritis (JIA). Our study is aimed to evaluate the possible difference in arthritis course depending on uveitis presence in patients with JIA, treated with biologics.

**Methods:**

From our database of patients with JIA treated with biologics, we extracted patients to whom the first agent was administrated with or without MTX. The exclusion criteria included treatment with current systemic corticosteroids, infliximab, rituximab, observation period <3 years, and no missing data. After selection, 175 patients were eligible for analysis. We evaluated clinically significant flare with joint involvement (which required change of biologic or non-biologic DMARD) and time to flare. We compared two groups: (i) patients with uveitis (*n* = 32) and (ii) patients without uveitis (*n* = 143). For statistical analysis, we used Cox's regression models, the log-Rank test, *x*^2^ test, and the Mann–Whitney test.

**Results:**

There was no difference in gender distribution and achievement of arthritis remission between groups. Patients in the non-uveitis group predominantly received etanercept (64.3%). In the uveitis group, the most prescribed biologic agent was adalimumab (71.9%). The presence of uveitis increased the risk of JIA flare, OR = 3.8 (95% CI: 1.7; 8.7), and the cumulative probability of joint flare, RR = 4.5 (95% CI: 1.7; 12.1), *p* =.003, after adjustment on methotrexate, RR = 3.1 (1.6; 6.), *p* =.0008. In the subgroup of patients treated with adalimumab, the absence of methotrexate increased the cumulative probability of flare [RR = 6.5 (95% CI: 1.4; 31.1), *p* = 0.02].

**Conclusion:**

The presence of uveitis proved to be a risk factor in JIA flare. Methotrexate can decrease the cumulative flare probability. Further trials are required.

## Introduction

Juvenile idiopathic arthritis (JIA) is the most common pediatric rheumatic disease (1). JIA includes a heterogeneous group of chronic arthritis of unknown etiology that begins before 16 years (1). Uveitis is the most frequent extra-articular manifestation of JIA (2). JIA may have an unfavorable prognosis, leading to musculoskeletal system disability or visual impairment if adequate treatment is not prescribed (3–5). There are known predictors of uveitis in JIA, such as female gender, younger-onset age, ANA-positivity, oligoarticular course, and high CRP, but data about the features of the arthritis course in patients with concomitant uveitis are scarce (6, 7). Biological treatment is an option if previous therapy with methotrexate was ineffective or intolerable and can modify the course of arthritis and uveitis and their outcomes (8–10). The question about the benefits of combined treatment with biologics and methotrexate is still open. Meanwhile, the number of patients with JIA treated with biologics alone is growing due to methotrexate side effects correlated with treatment duration.

Most current studies aim to identify predictors of the development of uveitis in patients with JIA and its course, but there are limited studies to answer whether the presence of uveitis affects the severity of arthritis.

## Materials and Methods

### Study Design and Patients

Written consent was obtained according to the Declaration of Helsinki. The local ethics Committee of Saint-Petersburg State Pediatric Medical University approved the protocol of this trial (protocol # 3/11 from 04.12.2012).

The study population was selected retrospectively from the database of patients with JIA treated with biologics. The 1997 International League of Rheumatology Association (ILAR) criteria for JIA subgroups were used to diagnose JIA and determine the type of arthritis (11). Diagnosis of uveitis and its anatomic classification (anterior, intermediate, posterior, or panuveitis) was performed according to the Standardization of Uveitis Nomenclature Workshop (SUN) criteria (12).

### Inclusion Criteria

The inclusion criteria were as follows: (i) patients for whom the first biologic agent was administrated with or without methotrexate MTX; (ii) patients under a minimal observation period after biologic administration of 3 years; (iii) patients who developed uveitis *de novo* during the observation period were eligible only for group “uveitis” since uveitis was diagnosed.

### Exclusion Criteria

The exclusion criteria were as follows: (i) systemic-onset JIA; (ii) acute anterior uveitis; (iii) treatment with current systemic corticosteroids, infliximab, and rituximab; and (iv) patients observed in our center irregularly or for <3 years. After selection, 175 patients were eligible for analysis.

### Patient's Demographics

In each patient, the following were evaluated: (i) clinical and demographic data: gender, age of the JIA onset, JIA category, and number of active joints; (ii) laboratory data: C-reactive protein (CRP), erythrocyte sedimentation rate (ESR), and antinuclear antibodies (ANA)-positivity; and (iii) treatment options: methotrexate (date of initiation and current status, whether the patient receives methotrexate or not), biologic agent (type, date of administration). The presence of uveitis was examined by an experienced ophthalmologist (TN, 30 years in uveitis), with the slit-lamp examination.

### Study Outcomes

In each patient, we evaluated if a clinically significant flare with joint involvement (which required a change of biologic or non-biologic DMARD) occurred or not and the time to flare. The patients were divided into two groups: (i) patients with uveitis (*n* = 32) and (ii) patients without uveitis (*n* = 143). At least, a 3-year observation period was required to accurately select between “uveitis” and “no uveitis” groups because uveitis usually develops within the first 2 years after the JIA onset.

### Statistical Analysis

Each quantitative variable was checked with the Kolmogorov–Smirnov test, and no normal distribution was found. Descriptive statistics were reported in medians and interquartile ranges (IQRs) for continuous variables and absolute frequencies and percentages for categorical variables. We utilized the Mann–Whitney *U*-test to compare quantitative variables in two groups and the chi-square test to compare qualitative data, or the Fisher's exact test in case of expected frequencies <5. Odds ratio (OR) with a 95% confidence interval (CI) was calculated to assess the risk of JIA flares. Survival analysis with arthritis flare as the event of interest was conducted using the Kaplan–Meier method. The log-rank test compared survival curves. A Cox proportional hazards regression model tested factors significantly associated with time to achievement ID status and flares. A *p* < 0.05 was considered statistically significant. The software Statistica (release 10.0, StatSoft Corporation, Tulsa, OK, USA) was used for data analysis. The *p* < 0.05 were considered to indicate a significant difference.

## Results

The uveitis group has the following typical characteristics: younger age, less active joints, and ANA-positivity compared to the non-uveitis group. The female gender, ESR, CRP, and remission frequency were equal between the two groups. The patients with uveitis had more frequent flares during the observation period and a shorter time before the first significant flare (Table 1).

**Table 1:** The main demographic parameters of the study population.

**Parameters**	**Uveitis (*n* = 32)**	**No uveitis (*n* = 143)**	***p***
Onset age, years, Me (25–75%)	3.9 (2.1; 6.2)	5.5 (2.5; 10.5)	0.033
Sex, women	21 (65.6)	95 (66.4)	0.930
ANA positivity, *n* (%)	15/26 (57.7)	26/72 (36.1)	0.056
ESR, mm/h, Me (25–75%)	12,5 (7.0; 19.0)	10.0 (4.0; 20.0)	0.406
CRP, mg/l, Me (25–75%)	2.2 (0.5; 6.2)	1.5 (0.4; 7.7)	0.593
Active joints, Me (25–75%)	1.0 (1.0; 2.0)	4.0 (2.0; 8.0)	0.000005
Oligoarthritis, *n* (%)	28 (87.5)	84 (58.7)	0.002
Ongoing methotrexate, *n* (%)	26 (81.3)	103 (72.0)	0.284
Remission, *n* (%)	23 (71.9)	101 (70.6)	0.889
First significant flare, *n* (%)	14 (43.8)	24/142 (16.9)	0.0009
Time to first significant flare, months, Me (25–75%)	33.0 (16.7; 61.5)	48.6 (26.4; 65.7)	0.023
Switching of the first biologic, *n* (%)	7 (21.9)	18/142 (12.7)	0.180
Time to switch of the 1st biologics, months, Me (25–75%)	49.0 (24.5; 62.1)	52.3 (35.0; 66.2)	0.186

*ANA, antinuclear antibodies; CRP, C reactive protein; ESR, erythrocyte sedimentation rate; Me, median*.

### Treatment

Etanercept (64.3%) was the main biological drug in the non-uveitis group and adalimumab (71.9%) in the uveitis group. Remission was observed in ~70% of the patients in both groups—seven children from the uveitis group and 18 from the non-uveitis group required the first biological drug change. In the uveitis group, adalimumab switched to infliximab and *vice versa*. In the non-uveitis group, etanercept was switched to adalimumab or tocilizumab.

### Flare Risk Factors

**Figure 1:**
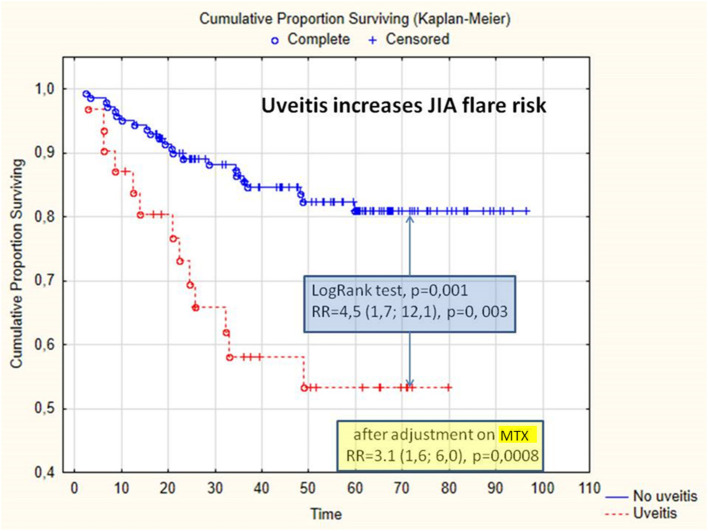
Cumulative proportion of survival without arthritis flare, depending on the presence of uveitis. JIA, juvenile idiopathic arthritis; MTX, methotrexate; RR, relative risk.

Uveitis increases the risk of JIA flare, OR = 3.8 (95% CI: 1.7; 8.7), and the cumulative probability of flare, RR = 4.5 (95% CI: 1.7; 12.1), *p* = 0.003; log-rank test, *p* = 0.001 (Figure 1). After adjusting methotrexate treatment, uveitis is a risk factor in arthritis flare, RR = 3.1 (1.6; 6.), *p* = 0.0008. In the subgroup of the patients treated with adalimumab, the absence of methotrexate increases the cumulative probability of arthritis flare, RR = 6.5 (95% CI: 1.4; 31.1), *p* = 0.02; in the patients treated with adalimumab, uveitis increases the cumulative probability of arthritis flare.

## Discussion

Uveitis might be a risk factor of more severe course of JIA. In previous studies, it was shown that the JIA category, e.g., oligoarthritis, is an independent risk factor of uveitis when combined with ANA-positivity and early age of the JIA onset (13–15). It is well-established that persistent oligoarthritis and extended oligoarthritis have the highest probability of developing uveitis, while, in systemic and RF-positive categories, uveitis occurs extremely rarely (5, 14, 15). The risk factors in acute anterior uveitis in enthesitis-related arthritis are male gender, ANA-positivity, and HLA B27 (2, 16, 17). The role of uveitis in the course of arthritis has not been previously described. Uveitis and arthritis might have similar pathogenesis related to immune cell activation and TNF-a hyperproduction (18, 19). Both arthritis and uveitis respond well to methotrexate and TNF-a inhibitors (adalimumab and infliximab) (20). Earlier prescription of methotrexate and biologics may decrease the incidence of uveitis (21–23). The combination of biologic and non-biologic DMARDs reduces the probability of active uveitis at any instance in time (22). The arthritis flare has a temporal association with uveitis flare (22). In our study, the absence of methotrexate increased the cumulative likelihood of an exacerbation rate in the subgroup of the patients receiving adalimumab. Immunogenicity of biologics, especially adalimumab, was associated with the production of antibodies against biologics. The protective role of methotrexate against the production of anti-adalimumab antibodies leads to preserving serum concentration of adalimumab and its efficacy (24, 25). The treatment efficacy of JIA-associated uveitis and arthritis with a combination of biologic and methotrexate has already been demonstrated in several studies (26–29).

According to our experience, we recommend starting methotrexate treatment early in patients with high risks of uveitis as well as continuing methotrexate in patients with JIA and uveitis, especially those who are treated with adalimumab, despite intolerance or adverse events occur.

The limitations of the study are related to its retrospective nature, the variation in times of uveitis development, and the absence of standard treatment protocol throughout the study. The use of various biologics may also influence the results. Different types of immunogenicity of adalimumab and etanercept may introduce bias into the results.

## Conclusion

The results of our analysis indicate the need for ongoing cooperation between a pediatric rheumatologist and an ophthalmologist. Uveitis might be a hallmark of arthritis flare, and methotrexate therapy can reduce the likelihood of arthritis flare.

## Data Availability Statement

The original contributions presented in the study are included in the article/supplementary material, further inquiries can be directed to the corresponding author/s.

## Ethics Statement

The studies involving human participants were reviewed and approved by The local Ethics Committee which approved the protocol of this trial of Saint-Petersburg State Pediatric Medical University (protocol # 3/11 from 04.12.2012). Written informed consent to participate in this study was provided by the participants' legal guardian/next of kin.

## Author Contributions

MK, EG, LSSo, and IA designed the study. MK, EG, LSSo, IA, TN, EI, IC, YK, EO, LSSn, VM, MD, OK, and VC collected and analyzed the data. MK, EG, IA, and LSSo wrote the manuscript. All authors contributed to manuscript revision, read, and approved the submitted version.

## Funding

This work was supported by Russian Science Foundation Grant 20-45-01005.

## Conflict of Interest

The authors declare that the research was conducted in the absence of any commercial or financial relationships that could be construed as a potential conflict of interest.

## Publisher's Note

All claims expressed in this article are solely those of the authors and do not necessarily represent those of their affiliated organizations, or those of the publisher, the editors and the reviewers. Any product that may be evaluated in this article, or claim that may be made by its manufacturer, is not guaranteed or endorsed by the publisher.
